# Management of dermatomyositis patients amidst the COVID-19 pandemic: Two case reports

**DOI:** 10.1097/MD.0000000000030634

**Published:** 2022-09-23

**Authors:** Yumeng Cao, Jingrun Zhou, Tingting Cao, Guqin Zhang, Huaqin Pan

**Affiliations:** a Department of Respiratory and Critical Care Medicine, Zhongnan Hospital of Wuhan University, Wuhan, Hubei Province, China; b Department of Gastroenterology, Zhongnan Hospital of Wuhan University, Wuhan, Hubei Province, China; c Department of Critical Care Medicine, Zhongnan Hospital of Wuhan University, Wuhan, China; d Clinical Research Center of Hubei Critical Care Medicine, Wuhan, Hubei Province, China

**Keywords:** case report, coronavirus, COVID-19, dermatomyositis, pandemic, SARS-CoV-2 virus

## Abstract

In December 2019, a new epidemic of coronavirus disease 2019 (COVID-19) appeared in Wuhan, Hubei Province, and spread rapidly to other parts of China and worldwide. Although established methods exist for the diagnosis and treatment of COVID-19 infection, the management of dermatomyositis (DM) patients with COVID-19 is unknown.

**Patient concerns:**

In this article, we describe case reports of 2 patients with DM. The first case was a 67-year-old patient with DM and infected with COVID-19 who was admitted to Leishenshan Hospital for a 1-month history of fever, cough, and expectoration. The second case was a 51-year-old male patient who was admitted to Leishenshan Hospital due to fever with cough, expectoration and shortness of breath for 1 month.

**Diagnoses:**

The first patient was diagnosed with COVID-19 secondary to DM based on repeated SARS-CoV-2 real-time reverse-transcriptase polymerase-chain-reaction (RT-PCR) test, detailed medical history and chest computed tomography; The second patient was diagnosed with interstitial lung disease associated with anti-MDA5 DM based on the results of antirheumatic and anti-inflammatory therapy and the above 3 methods.

**Interventions and outcomes:**

The first patient received supportive and empirical treatment, including antiviral treatment, anti-inflammatory treatment, oxygen therapy and prophylactic anticoagulation therapy. The symptoms and laboratory results got improved after the treatments. He was discharged with thrice negative PCR tests for the SARS-CoV-2 virus. The second patient received a comprehensive treatment, including glucocorticoid and plasma exchange; his symptoms were relieved and improved.

**Lessons:**

These cases suggest that repeated new pathogenic test results for the coronavirus and a detailed diagnosis of the medical history are important means to distinguish these diseases. Increased attention to the individual characteristics of different cases may allow for more effective diagnosis and treatment.

## 1. Introduction

In December 2019, a new epidemic of coronavirus disease 2019 (COVID-19) caused by the severe acute respiratory syndrome coronavirus 2 (SARS-CoV-2) appeared in Wuhan, Hubei Province, China. COVID-19 is highly infectious through respiratory droplets and direct and indirect contact via surfaces^[1]^; therefore, it spread rapidly to other parts of China and worldwide.^[2]^ Globally, as of 6:36 pm CEST, August 12, 2022, there have been 585,950,085 confirmed cases of COVID-19, including 6,425,422 deaths.^[3]^ The most common symptoms at the onset of illness in COVID-19 patients are respiratory compromise with fever and cough, dyspnea, myalgia, or fatigue. The most common radiologic patterns on chest computed tomography (CT) were ground-glass opacity (GGO) and bilateral patchy shadowing.^[4]^

Dermatomyositis (DM) is a multisystem autoimmune disease caused by a complex interplay of genetics and environmental exposure with various influencing factors. Lung involvement is frequently observed in patients with DM. Dyspnea and nonproductive cough are the most common symptoms in these patients. The chest CT in DM patients shows GGO, honeycombing, or reticular shadows.

Although there are methods for the diagnosis and treatment of COVID-19 infection, the clinical characteristics, treatment, and prognosis of patients with DM disease and those infected with COVID-19 are not well established. Here we report 2 DM cases that may provide a basis for the clinical treatment of COVID-19-infected DM patients.

## 2. Case presentation

### 2.1. Case 1

#### 2.1.1. Chief complaints.

A 67-year-old male patient living in Wuhan was admitted to Leishenshan Hospital for a 1-month history of fever, cough, and expectoration.

#### 2.1.2. History of present illness.

The patient had suffered from fever, cough, and expectoration for a month, without myalgia, dyspnea, chills, diarrhea, nausea, vomiting or hemoptysis. Before admission, the patient had been diagnosed with COVID-19 with twice positive real-time reverse-transcriptase polymerase-chain-reaction (RT-PCR) tests for the SARS-CoV-2 virus. The patient had received symptomatic supportive therapy such as oxygen inhalation, antiviral, antibacterial, cough, and phlegm relief in the local hospital. However, the patient still had a cough and sputum.

#### 2.1.3. History of past illness.

His previous medical history was DM for >1 year with the treatment of prednisone (20 mg/day).

#### 2.1.4. Physical examination upon admission.

Physical examination revealed a body temperature of 36.5°C, a blood pressure of 130/80 mm Hg, a pulse of 75 beats per minute, and a respiratory rate of 18 breaths per minute.

#### 2.1.5. Laboratory examinations.

Laboratory results were summarized as follows:

- Lymphocyte count and percentage dramatically decreased.
- Thrombocyte count mildly decreased.
- Levels of alanine aminotransferase (ALT), aspartate aminotransferase (AST), D-dimer and erythrocyte sedimentation rate were noticeably elevated (Table 1).

**Table 1.** Clinical characteristics of the patient with dermatomyositis infected with COVID-19 in Case 1.

Measures	Normal range	Feb. 21 Day 3	Mar. 03 Day 14			Mar. 13 Day 24	Mar. 18 Day 29	
**Laboratory examinations**								
White blood cells, ×10^9/L	3.5-9.5	3.26		4.7	NA			3.39
Neutrophil counts, ×10^9/L	1.8-6.3	2.25		3.32	2.57			1.78
Lymphocyte counts, ×10^9/L	1.1-3.2	0.58		0.77	0.61			0.99
C-reactive protein, mg/L	0-10.0	-		5.69	2.96			1.84
D-dimer, mg/L	0-500	8.06		-	1.35			-
BUN, pg/mL	0-100	6.2		5.2	6.7			6.1
Cr, μmol/L	64-104	66.6		69.5	72.6			70.9
Uric acid, μmol/L	208-428	209		304	369			344
ESR, mm/H	0-20	25		27	31			-
IL-6, pg/mL	0-7	NA		NA	2.86			-
**Vital signs**								
Temperature, °C	36-37	37.9		36.5	36.6			36.5
Pulse, beats per minute	60-100	98		88	72			82
Respiratory rate, breaths per minute	16-20	20		21	19			17
Blood pressure, mmHg	90-140/60-90	126/77		115/83	118/78			122/86
**Treatments**								
	Low flow oxygen therapy							
	Antiviral therapy (abidol, ribavirin and Chinese medicine)							
	Prevent secondary infection (Moxifloxacin)							
	Liver function improvement and protection therapy (diammonium glycyrrhizinate enteric-coated capsules)							
	Prophylactic anticoagulation therapy (low molecular weight heparin)							
	Attenuate lung inflammation (methylprednisolone 40 mg, once daily, iv)							

BUN = blood urea nitrogen, Cr = creatinine, ESR = erythrocyte sedimentation rate, IL-6 = Interleukin-6, NA = none available.

#### 2.1.6. Imaging examinations.

**Figure 1.**
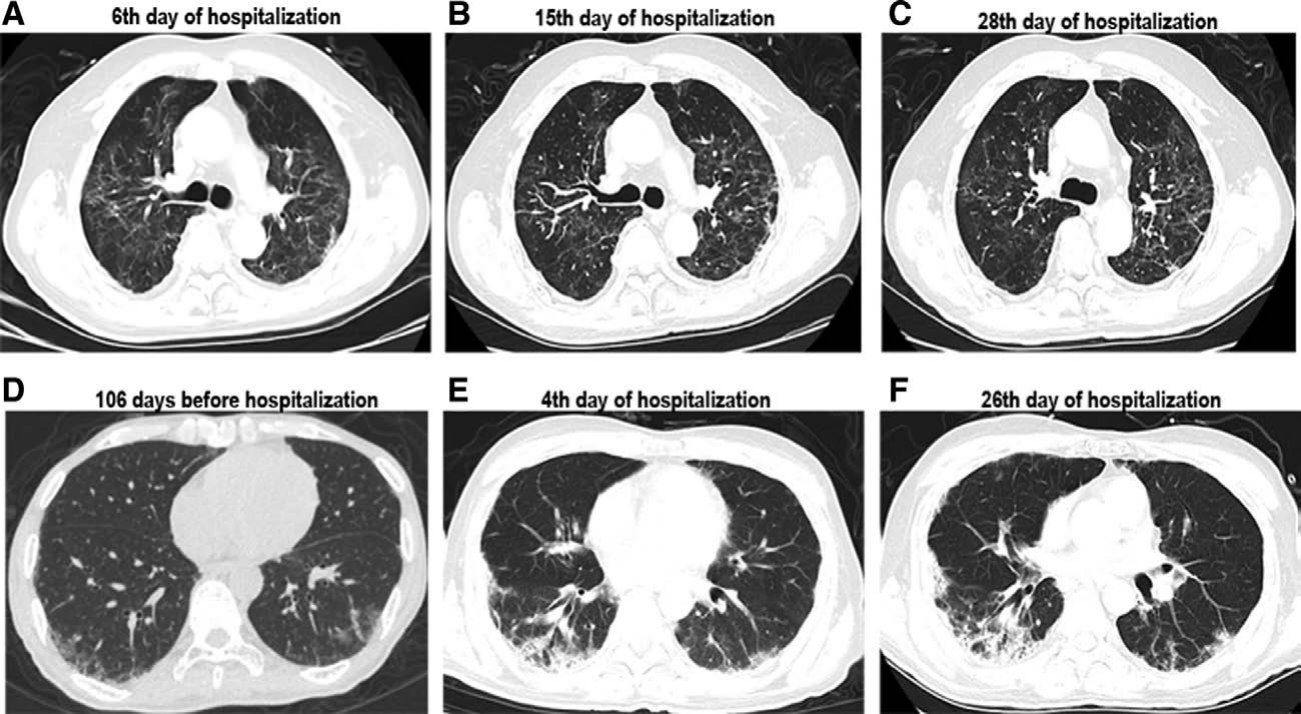
High-resolution computed tomography images during the disease course. (A) 6th day of hospitalization of case1 bilateral peripheral ground-glass opacification (GGO); (B) 15th day of hospitalization of case1 bilateral diffuse ground-glass shadow; (C) 28th day of hospitalization of case1 the shadow of GGO was mainly in the left lung lobe; (D) 106 days day before hospitalization of case 2 bilateral interstitial infiltrating shadows; (E) 4th day of hospitalization of case 2 right subpleural patchy shadow and bilateral ground glass shadow; (F) 26th day of hospitalization of case 2 right subpleural honeycomb shadow and bilateral ground glass shadow.

The chest CT on hospital day 6 showed bilateral peripheral ground-glass opacification (GGO) (Fig. 1).

#### 2.1.7. Further diagnostic work-up.

The patient was further evaluated with blood cultures, urine, and stool cultures which turned out negative. The patient tested positive for SARS-CoV-2 virus by PCR test twice.

### 2.2. Final diagnosis

The final diagnosis of the presented case is COVID-19 pneumonia.

### 2.3. Treatment

Treatment during hospitalization was largely symptomatic and supportive. The patient was supplied with persistent low flow oxygen therapy, abidol (0.1g, twice daily, po), ribavirin (0.5g, once daily, iv), and Chinese medicine such as antiviral therapy. Moxifloxacin (0.4 g, once daily, po) was also administered to prevent secondary infection, as well as diammonium glycyrrhizinate enteric-coated capsules (150mg, thrice daily, po) for liver function improvement and protection therapy and low-molecular weight heparin (LMWH) as prophylactic anticoagulation therapy. To attenuate lung inflammation, a low dose of methylprednisolone (40 mg, once daily, iv) was administered.

The patient’s symptoms continued to alleviate with active treatments. Amounts of methylprednisolone were gradually decreased and discontinued on day 11 of hospitalization. LWMH was discontinued on day 32 of hospitalization.

### 2.4. Outcome and follow-up

With treatment, the patient was asymptomatic aside from an intermittent dry cough. The previous GGO in both lungs was alleviated, and the results of laboratory reexamination were significantly improved. When the patient tested negative for SARS-CoV-2 virus by PCR test thrice, he was discharged and remained in quarantine for 14 days.

### 2.5. Case 2

#### 2.5.1. Chief complaints.

A 51-year-old man was admitted to Leishenshan Hospital due to fever with cough, expectoration, and shortness of breath for 1 month.

#### 2.5.2. History of present illness.

The patient’s symptoms started a month ago. Nucleic acid detection of COVID-19 was negative in the local hospital prior to admission. He was suspected to be infected with the SARS-COV-2 virus.

#### 2.5.3. History of past illness.

The patient was diagnosed with interstitial lung disease related to anti-MDA5 DM in November 2019 and received glucocorticoid therapy.

#### 2.5.4. Physical examination upon admission.

The physical examination revealed a body temperature of 36.8°C, a blood pressure of 116/83 mm Hg, a pulse of 124 beats per minute, a respiratory rate of 20 breaths per minute, and oxygen saturation of 94% without oxygen inhalation.

#### 2.5.5. Laboratory examinations.

Blood analysis revealed a mild leukocytosis of 3.67 × 10^9^/L, with predominant neutrophils (3.10 × 10^9^/L). Serum C-reactive protein was increased at 20.63 mg/L (normal range < 8 mg/L) (Table 2).

**Table 2.** Clinical characteristics of the patient with dermatomyositis pulmonary interstitial fibrosis in Case 2.

Measures	Normal range	Mar.07 Day 2	Mar.9 Day 4	Mar.13 Day 8	Mar.15 Day 10	Mar.17 Day 12	Mar.19 Day 14	Mar.23 Day 18	Apr.01 Day 27
**Laboratory examinations**									
White blood cells, ×10^9/L	3.5-9.5	3.67	8.43	4.92	4.95	7.18	7.21	6.95	6.62
Neutrophil counts, ×10^9 /L	1.8-6.3	3.1	7.61	4.22	4.24	6.34	6.62	6.18	5.91
Lymphocyte counts, ×10^9/ L	1.1-3.2	0.33	0.45	0.38	0.51	0.48	0.33	0.4	0.36
C-reactive protein, mg/L	0-10.0	20.63	0.56	8.33	1.4	0.5	15.75	4.27	6.77
D-dimer, mg/L	0-500	1.1	1.24	0.95	-	-	-	-	-
BUN, pg/mL	0-100	10.54	131.5	22.73	33.73	11.89	-	-	-
Cr, μmol/L	64-104	53.4	68.3	44	46.4	46.1	45.3	43.5	51.3
Uric acid, μmol/L	208-428	172	149	114	127	173	201	169	146
ESR, mm/H	0-20	7	22	29	28	26	-	29	1.32
IL-6, pg/mL	0-7	55.34	376.9	2.95	-	9.97	26.74	3.98	-
**Vital signs**									
Temperature, °C	36-37	37.4	39.3	36.5	36.4	36.5	36.7	36.5	36.5
Pulse, beats per minute	60-100	-	-	-	-	-	-	-	-
Respiratory rate, breaths per minute	16-20	27	20	17	23	33	23	20	34
Blood pressure, mmHg	90-140/60-90	88/60	110/65	126/83	-	115/78	108/77	124/87	115/68
**Treatments**	Low flow oxygen therapy								
	Symptomatic and supportive treatment								
	Fluid resuscitation, vasopressor, shock therapy								
	Antiviral therapy								
	Prophylactic anticoagulation therapy (low molecular weight heparin)								
	Immunotherapy (tocilizumab)								
	Anti-inflammation (glucocorticoid 80 mg, once daily, iv)								
	Plasma exchange								

BUN = blood urea nitrogen, Cr = creatinine, ESR = erythrocyte sedimentation rate, IL-6 = Interleukin-6.

#### 2.5.6. Imaging examinations.

Chest CT imaging showed bilateral interstitial infiltrating shadows (Fig. 1).

#### 2.5.7. Further diagnostic work-up.

The anti-MDA5 antibody was positive, and the repeated PCR test for the SARS-CoV-2 virus was negative.

### 2.6. Final diagnosis

The final diagnosis of the presented case is interstitial lung disease (ILD) associated with anti-MDA5 DM, rather than COVID-19 pneumonia.

### 2.7. Treatment

An hour and a half after admission, the patient suddenly developed dyspnea with a declining blood pressure of 88/60 mm Hg, as well as tachycardia. He was supplied immediately with rehydration, anti-shock, active vascular medicine, anti-virus, and symptomatic and supportive treatment. Subsequently, he was transferred to the intensive care unit. The risk score of deep vein thrombosis was 5 and LMWH was administered to prevent thrombosis. However, the patient’s state did not improve despite active treatments. Results of the pharyngeal swab specimen, nasal swab specimen and anal swab specimen analysis by SARS-CoV-2 RT-PCR test confirmed the patient to be negative for COVID-19. The levels of anti-SS-A/Ro 52KD antibody and anti-SS-A/Ro 62KD antibody were markedly increased. The final diagnosis was IDL (severe). He was supplied with tocilizumab, glucocorticoid (80 mg, once daily, iv) and plasma exchange (Table 2). After treatments, the patient’s symptoms continued to alleviate.

### 2.8. Outcome and follow-up

After 30 days of treatment, the results of continuous laboratory examination showed that abnormal indicators such as interleukin-6 (IL-6) returned to normal (Table 2). CT dynamic monitoring showed that symptoms were relieved and improved compared with symptoms at the time of admission (Fig. 1). The patient was discharged on April 4 and transferred to a local hospital.

## 3. Discussion

The lung is a frequently damaged organ of autoimmune-mediated injury in patients with DM.^[5,6]^ DM patients with lung involvements usually present with interstitial lung disease.^[7,8]^ Patients may have a dry cough, fever, and dyspnea. The most common presentation of COVID-19 is fever, cough and respiratory tract infection.^[9]^ The high similarity of these clinical features between the 2 diseases presented a great challenge to accurate diagnosis and pinpoint management during the COVID-19 pandemic. Patients with DM have a high risk of infection by SARS-COV-2. The management of COVID-19 patients with DM is not well established. Multiple research studies have found that comorbidities may increase the risk of COVID-19 patients.^[10,11]^ Patients with DM are often immunosuppressed or immunocompromised due to the long-term use of corticosteroids and/or immunosuppressive agents.^[12]^ Bonometti et al^[13]^ reported the first case of systemic lupus erythematosus (SLE) triggered by COVID-19 infection. Kogami et al^[14]^ reported a case of a patient with interstitial pneumonia developing as a complication of anti-MDA5 antibody-positive DM diagnosed with COVID-19 during remission induction therapy. These indications may have unique clinical manifestations when combined with COVID-19; therefore, increased attention should be paid to the clinical management of such patients. In the second case we reported, the patient was diagnosed with COVID-19 combined with DM on admission to hospital for typical respiratory symptoms. The patient responded significantly worse than expected with aggressive treatment. Repeated and multi-regional nucleic acid detections of COVID-19 were negative. Finally, we considered that the diagnosis of this patient was interstitial lung disease associated with anti-MDA5 DM, rather than 2019 coronavirus disease pneumonia. This deduction was substantiated by the results of antirheumatic and anti-inflammatory therapy. These case reports demonstrate that properly managing of COVID-19 infection is crucial for distinguishing between viral infection and autoimmunity. It is highly important that nucleic acid detections with COVID-19 are considered in distinguishing lung involvements of DM and COVID-19.

The treatment of patients with COVID-19 secondary to DM is also significantly different from patients with COVID-19 or DM. In the first case we reported, the patient gave priority to antiviral therapy and was assisted with systemic corticosteroid treatment. The second patient presented more seriously and received corticosteroid impulse and plasmapheresis therapy. The treatment of interstitial lung disease associated with DM mainly includes immunosuppression, physical therapy, monitoring, and prevention of complications.^[15]^ Drug treatment includes corticosteroids, azathioprine, cyclophosphamide, methotrexate, biologic agents, and so on.^[5]^ Corticosteroids are considered the mainstay of clinical management and first-line therapy of interstitial lung disease associated with DM.^[16–18]^ If treatments continue to be ineffective after 3 to 6 months of corticosteroids, or if the patient relapses, another immunosuppressant is usually added. Corticosteroids are not recommended as a priority treatment in the management of COVID-19. The role of corticosteroids is highly disputed to treat severe infections.^[19–21]^ Guidelines from the World Health Organization (WHO) do not recommend reliance on corticosteroids as a routine treatment for the clinical management of COVID-19.^[22]^ Corticosteroids may inhibit immune responses and pathogen clearance when suppressing lung inflammation and could lead to delayed viral clearance and worsened outcomes. A systematic review and meta-analysis of 6548 patients found that the use of corticosteroids was associated with higher mortality and a higher rate of secondary infection.^[23]^ Conversely, a recent meta-analysis of 7 randomized trials indicated that compared with usual care, systemic corticosteroids reduced mortality in critically ill hospitalized patients with COVID-19.^[24]^ According to clinical experience, we hold the opinion that low-dose corticosteroids are effective and safe for severe and critical patients and may play a role in improving the prognosis of COVID-19 patients with DM. Further large-scale studies are required to confirm these findings. In compiling a treatment plan, the advantages and disadvantages of corticosteroids should be taken into consideration.

## 4. Conclusions

This is the first article to discuss patients with DM infected by the SARS-COVID-2 and patients with interstitial pneumonia secondary to DM. Although they have similar clinical manifestations, CT scans and laboratory test results, they are very different concerning clinical treatment. Repeated SARS-CoV-2 RT-PCR test and a detailed diagnosis based on medical history are greatly important to distinguish the 2 diseases. Effective treatment should be carried out according to the individual disease characteristics of different cases.

## Acknowledgement

We thank Emily Pellegrini from Dascena (San Francisco, CA, USA) for the language editing.

## Author contributions

HQP and GQZ conceived the research, YMC and JRZ designed the study, interpreted data, and wrote the manuscript. TTC contributed to the acquisition of clinical data. All authors contributed to the article and approved the submitted version.

**Conceptualization:** Guqin Zhang, Huaqin Pan.

**Data curation:** Guqin Zhang, Huaqin Pan.

**Formal analysis:** Jingrun Zhou, Yumeng Cao.

**Investigation:** Tingting Cao, Yumeng Cao.

**Methodology:** Jingrun Zhou, Yumeng Cao.

**Supervision:** Guqin Zhang, Huaqin Pan.

**Validation:** Yumeng Cao.

**Writing – original draft:** Jingrun Zhou, Yumeng Cao.

**Writing – review & editing:** Guqin Zhang, Huaqin Pan.
